# Validity of two subjective skin tone scales and its implications on healthcare model fairness

**DOI:** 10.1038/s41746-025-01975-7

**Published:** 2025-10-03

**Authors:** Cassandra W. Cu, Nicole E. Dundas, Timothy Heintz, Zahida A. Sheikh, Bianca Alonso-Bermudez, Jasmine Walker, Avery Wooten, Anusha Badathala, Allyson Chapman, Odinakachukwu Ehie, Karthik Raghunathan, Hunter Mills, Edie Espejo, John Boscardin, Arthur W. Wallace, Julien Cobert

**Affiliations:** 1 School of Medicine, Tufts University School of Medicine, Boston, MA USA; 2 UC Berkeley Department of Bioengineering, Berkeley, CA USA; 3 Department of Anesthesiology, Perioperative and Pain Medicine, Brigham and Women’s Hospital, Boston, MA USA; 4 School of Medicine, University of California, San Francisco, San Francisco, CA USA; 5 Division of Anesthesia, San Francisco Veterans Affairs Medical Center, San Francisco, CA USA; 6 Critical Care and Palliative Medicine, Department of Internal Medicine, University of California San Francisco, San Francisco, CA USA; 7 Department of Surgery, University of California San Francisco, San Francisco, CA USA; 8 Department of Anesthesia and Perioperative Care, University of California San Francisco, San Francisco, CA USA; 9 Department of Anesthesia and Perioperative Care, Duke University, Durham, NC USA; 10 Bakar Computational Health Sciences Institute, University of California, San Francisco, CA USA; 11 Division of Geriatrics, Department of Medicine, University of California, San Francisco, San Francisco, CA USA

**Keywords:** Interdisciplinary studies, Health care, Health services

## Abstract

Skin tone assessments are critical for fairness evaluation in healthcare algorithms (e.g., pulse oximetry) but lack validation. Using prospectively collected facial images from 90 hospitalized adults at the San Francisco VA, three independent annotators rated facial regions in triplicate using Fitzpatrick (I–VI) and Monk (1–10) skin tone scales. Patients also self-identified their skin tone. Annotator confidence was recorded using 5-point Likert scales. Across 810 images in 90 patients (9 images each), within-rater agreement was high, but inter-annotator agreement was moderate to low. Annotators frequently rated patients as darker when patients self-identified as lighter, and lighter when patients self-identified as darker. In linear mixed-effects models controlling for facial region and annotator confidence, darker self-reported skin tones were associated with lighter annotator scores. These findings highlight challenges in consistent skin tone labeling and suggest that current methods for assessing representation in biosensor-based algorithm studies may be influenced by labeling bias.

## Introduction

Predictive models, whether based on biosensor data or artificial intelligence (AI), are increasingly used in healthcare given its ability to achieve robust and accurate predictions from complex and heterogeneous data^1^. However, biases in these models can potentiate health disparities in vulnerable minority, demographic and socioeconomic groups^2–4^. For example, pulse oximeters, used to estimate blood oxygen levels, may overestimate blood saturation in individuals with darker skin tones, leading to delays in clinical interventions and increased mortality^5^. In dermatology, predictive models for skin lesion detection demonstrate significant disparities, with reduced accuracy in patients with darker skin tones due to poor representation in training datasets, leading to delays in melanoma diagnosis and worse outcomes^6,7^. Ensuring safety and fairness in predictive models requires that outputs do not result in differential accuracies, errors, or harms across sociodemographic characteristics and skin tones.

A primary method for evaluating bias in predictive models, particularly in computer vision and biometric devices, is to assess performance across different pigmentations^7,8^. More objective assessments of melanin content like reflectance spectrophotometry may be more accurate but cannot be performed post-hoc, as they require specialized equipment and are infeasible at scale. Other metrics like individual typology angle^9^ can be inconsistent and provide imperfect estimations of skin tone^10^. Subjective assessments used in clinical practice, most notably the Fitzpatrick scale^11^, have become the standard for skin tone classification. Originally developed in 1975 to assess UV sensitivity, the Fitzpatrick scale lacked a visual component^12,13^. Over time, adaptation into a perceived Fitzpatrick scale broadened its application beyond its original intent of skin tone classification for UV therapy dosing^14,15^. However, its widespread use is tempered by limitations in comprehensiveness and susceptibility to bias^7,15^. Google (Alphabet) has recently adopted the Monk Scale^16^ – created to be more inclusive of diverse skin tones– for more equitable results in search and image tools^17^. Yet, this scale still requires further validation from diverse groups^15^.

In this prospective study, we evaluated the reliability of skin tone classification across two scales, Fitzpatrick and Monk, by comparing assessments from three annotators and patients’ self-reporting. We hypothesized that skin tone classifications would be consistent within and across annotators and align with patient self-reported scores. Establishing robust, validated skin tone scales is crucial for dermatologic evaluations and for ensuring fairness and inclusivity in algorithmic tools such as facial image-based diagnostics and representation audits, including those assessing disparities in pulse oximetry performance.

## Results

### Characteristics of study sample

**Fig. 1:**
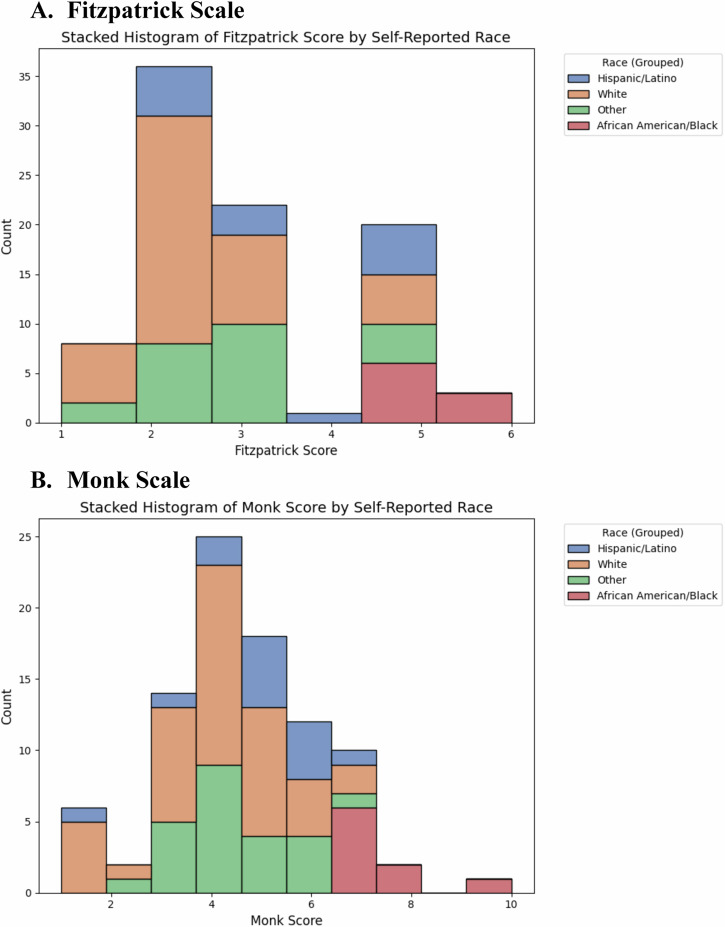
Distribution of Patient Self-Reported Skin Tone Scores. **A**, **B** – Distribution of Patient Self-Reported Skin Tone Scores. Histograms displaying the distribution of patient self-reported scores for Fitzpatrick (scale of I–VI) and Monk (scale of 1–10) across the study cohort. Most patients reported Fitzpatrick scores of II and Monk scores of 4.

Of the 130 enrolled in the parent study, 40 without Monk scores were excluded, yielding 90 participants for the study. Each annotator reviewed 810 total images (270 unique images across 3 sites each for 90 patients each in triplicate). The cohort was primarily male (77%), median age of 72 years (IQR: 59–76). Across participants, 48% self-identifying as White, 10% as African American/Black and 15.6% as Hispanic/Latino. Other race and ethnicity groups including Native Hawaiian/Pacific Islander, Multiracial, Asian, Native American/Alaska Native, Unknown, and Other (defined as “Other” to ensure anonymity^18^) were 26.3%. Participant demographics can be found in Supplementary Table 1. The distribution of self-reported scores across scales is shown in Fig. 1. Most patients self-reported as II on the Fitzpatrick scale and 4 on the Monk Scale.

### Internal rater reliability

Cronbach’s alpha values indicated high internal reliability among the annotators. At the patient level, the Fitzpatrick scale results had an alpha score ranging from 0.88 to 0.92, while Monk scale had similar alpha scores ranging from 0.88 to 0.93, across annotators shown in Supplementary Table 2. Analysis at the location level is in Supplementary Table 3.

### Inter-annotator agreement

For inter-annotator agreement in Table 1, our primary measure was the intraclass correlation coefficient (ICC[2,k]), based on a two-way random effects model, was 0.66 for Fitzpatrick (95%CI[0.02–0.87]) and 0.64 (95%CI[0.02–0.85]) for Monk. We conducted further sensitivity with the Weighted Cohen’s Kappa analysis on all the pairwise combinations, demonstrating agreement levels of 0.63 for Annotator 1 vs. 2, 0.39 for Annotator 1 vs 3, and 0.29 for Annotator 2 vs 3 for the Fitzpatrick scale and 0.64 for Annotator 1 vs. 2, 0.36 for Annotator 1 vs 3, and 0.30 for Annotator 2 vs 3 for the Monk scale. Using Kendall’s W to evaluate the ordinal relative rankings at the patient level across the annotators showed a score of 0.90 for the Fitzpatrick scale and 0.85 for the Monk scale. Krippendorf’s alpha was 0.41 for both scales.

**Table 1:** Inter-annotator agreement across skin tone scales

Analysis	Annotator pair	Fitzpatrick	Monk
Primary inter-annotator agreement measure			
Intraclass Correlation coefficient (ICC[2,k])	All annotators	0.66 (95% CI [0.02–0.87])	0.64 (95% CI [0.02–0.85])
Secondary inter-annotator agreement measures			
Weighted Cohen’s Kappa	1 vs. 2	0.63	0.64
	1 vs. 3	0.39	0.36
	2 vs. 3	0.29	0.30
Kendall’s W	All Annotators	0.90	0.85
Krippendorff’s Alpha	All Annotators	0.41	0.41

The Fitzpatrick (scale of I–VI) and Monk (scale of 1–10) refer to the two skin tone scales used for this study. Inter-rater reliability metrics comparing annotators’ ratings for Fitzpatrick and Monk scales. Weighted Cohen’s Kappa reflects pairwise agreement, Kendall’s W evaluates relative rankings across all annotators, Krippendorff’s Alpha measures ordinal agreement, and ICC[2,k] provides a measure of consistency across all annotators.

*ICC* intraclass correlation coefficient, *CI* confidence interval

### Comparing annotators and patient subjective scores

**Fig. 2:**
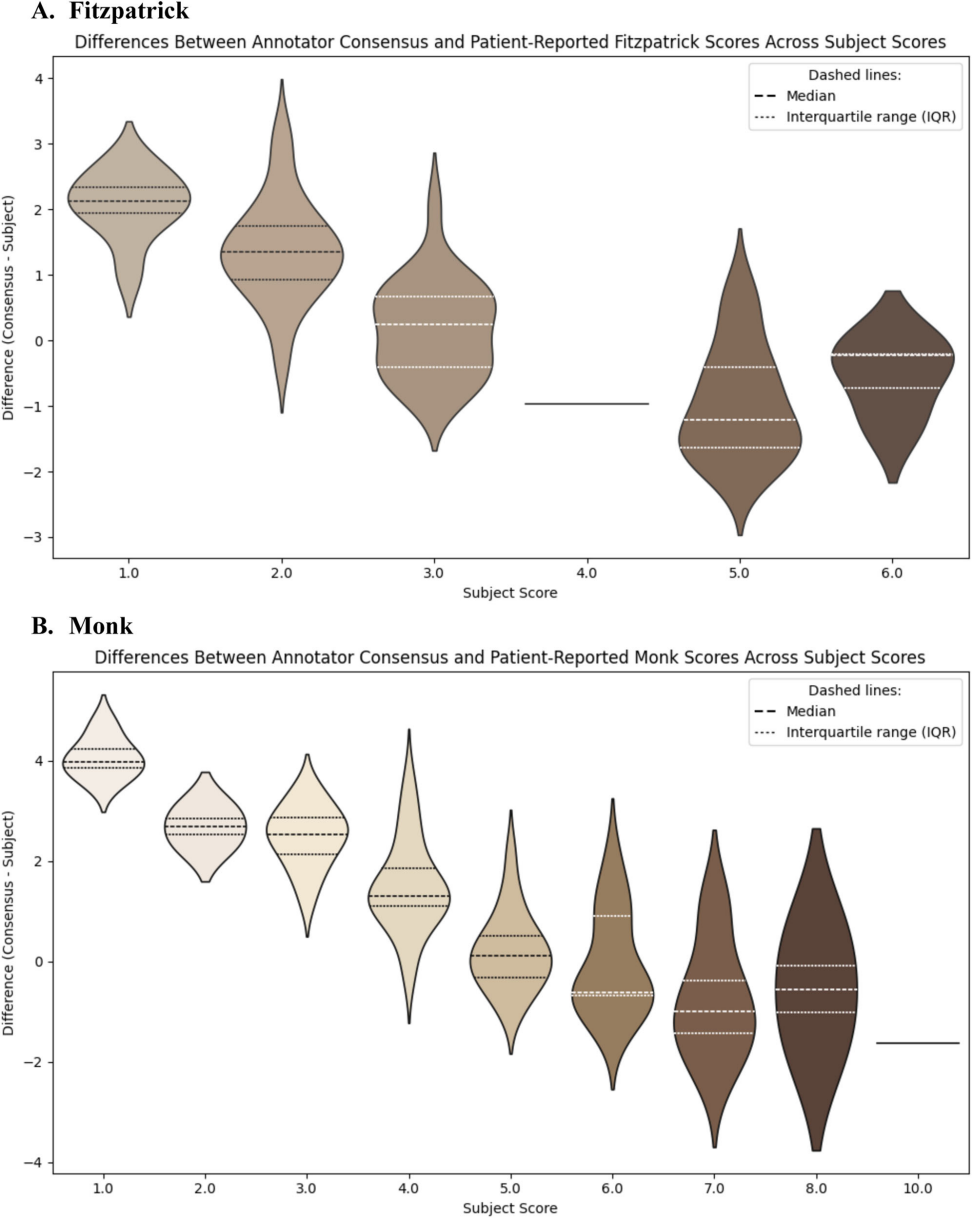
Violin Plots Comparing Annotator and Patient Scores. **A**, **B** – Violin Plots Comparing Annotator and Patient Scores. The Fitzpatrick (scale of I–VI) and Monk (scale of 1–10) refer to the two skin tone scales used for this study. Violin plot showing the distribution of differences between annotator consensus scores and patient self-reported scores, stratified by subject-reported scores. Positive values indicate that annotators assigned higher (darker) scores than the patients’ self-reported scores, while negative values indicate lower (lighter) annotator scores. The spread and median of differences highlight systematic discrepancies, with annotators tending to rate closer to the mid-range compared to the subject’s self-assessment.

A paired *t*-test comparing annotator consensus scores with patient self-reported skin tone scores showed statistically significant differences for Fitzpatrick and Monk (*p* < 0.001, Supplementary Table 4). Spearman’s correlation coefficients showed a strong negative correlation between the difference in annotator consensus scores and subjective scores vs. the subject scores themselves (−0.82 for Fitzpatrick and −0.84 for Monk; Supplementary Table 4). This relationship is visualized via a violin plot in Fig. 2A, B. The mixed linear model regression, controlling for facial location and annotator confidence, demonstrated that both higher self-reported Fitzpatrick and Monk scores were significantly associated with lower annotator scores (ß = −0.727, *p* < 0.001; ß = −0.823, *p* < 0.001, respectively) (Table 2). Annotator confidence levels of 4.0 and 5.0 for Fitzpatrick (ß = 0.157, *p* = 0.043; ß = 0.581, *p* < 0.001, respectively) and 3.0, 4.0, and 5.0 (ß = 0.723, *p* < 0.001; ß = 1.293, *p* < 0.001; ß = 1.726, *p* < 0.001, respectively) were significantly associated with higher annotator scores compared to baseline confidence levels of 1.0. Right cheek positions were associated with higher annotator scores compared to the forehead for both Fitzpatrick and Monk (ß = 0.385, *p* < 0.001; ß = 0.299, *p* < 0.001, respectively), while the left cheek showed no significant difference in either scale. Bland Altman Plots can be found in the Supplementary Fig. 3a, b.

**Fig. 3:**
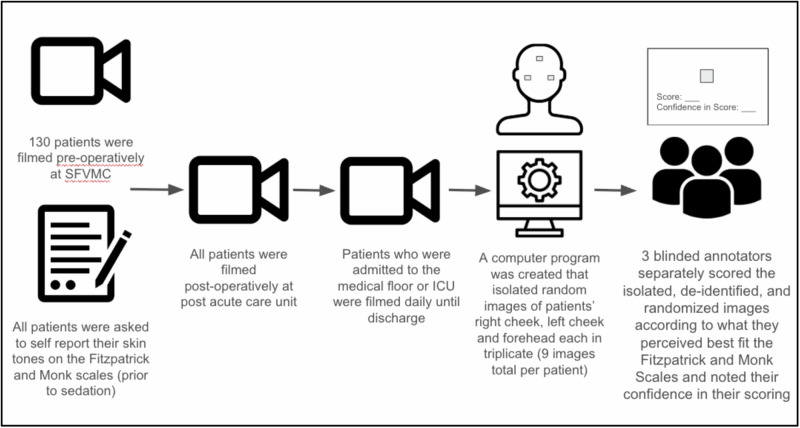
Data Pipeline and Protocol Flowchart. The data collected using the GUI was stored as separate spreadsheets for each annotator and each scale. Organizing this data starting with consolidating all of these spreadsheets into a Pandas Dataframe to start systemic evaluation across annotator ratings and scales. The patient subject data was also stored in a separate spreadsheet which was linked to each image using the common patient id number. All data organization and statistical analysis was conducted in Python. The Fitzpatrick (scale of I–VI) and Monk (scale of 1–10) refer to the two skin tone scales used for this study.

**Table 2:** Mixed linear model between the mean annotator score and patient self-reported scores

Variable	Fitzpatrick (ß, p)	Monk (ß, p)
Self-reported Score	−0.727 (*p* < 0.001)	−0.823 (*p* < 0.001)
Annotator Confidence 4.0	0.157 (*p* = 0.04)	1.293 (p < 0.001)
Annotator Confidence 5.0	0.581 (*p* < 0.001)	1.726 (*p* < 0.001)
Right Cheek Position	0.385 (*p* < 0.001)	0.299 (*p* < 0.001)
Left Cheek Position	0.057 (*p* = 0.20)	−0.028 (*p* = 0.53)

The Fitzpatrick (scale of I–VI) and Monk (scale of 1–10) refer to the two skin tone scales used for this study. Analysis of differences between annotator consensus score and patient self-reported skin tone scores for Fitzpatrick and Monk scales. Mixed linear models adjusted for image location and annotator confidence.

## Discussion

This study aimed to evaluate annotator reliability and agreement in skin tone classification across two commonly used skin tone scales and compare those scores to self-reported skin tones. Our findings highlight the importance of standardized guidelines in skin tone assessment to ensure consistency and reduce bias across methodologies. While internal reliability was high and annotators agreed on relative skin tone ordering, inter-annotator agreement was only moderate and highly variable. Annotator agreement was dependent on the individual annotators (moderate-low agreement between 1 and 2 and poor when including 3rd), suggesting individual annotator differences may play an important role. While future studies should increase the number of annotators to improve generalizability and robustness of our results, the inter-annotator variability calls into question the utility of subjective skin tone scales for fairness evaluation. More rigorous methodologies are required to support inclusive and accurate auditing in computer vision.

Our study adds to the growing literature highlighting inherent subjectivity in skin tone assessments. Previous groups have shown that our perception is influenced by individual and cultural experiences, and the scales themselves^8,19^. When comparing annotator consensus with patient-reported scores, significant discrepancies emerged. Mixed linear regression showed that annotators consistently assigned lighter skin tones than patients’ self-reports, with discrepancies varying by facial location, highlighting the need for clear annotation guidelines. Strong Spearman correlations indicated that annotators tended to rate patients with self-reported lighter skin tones higher, and those with darker tones lower. This suggests that patients often reported skin tones at the extremes of the scales, while annotators clustered ratings toward the middle.

Subjectivity and operator bias may influence Fitzpatrick and Monk scales, with individual annotators interpreting images in varied ways. These observations highlight the need for best practices in skin tone assessments, including multiple diverse annotators and clear protocols for disagreement resolution. The differences between annotator and patient scores suggest that self-reported and perceived external skin tones may be affected by patients’ internalized biases^20^, cultural context^21^, and social comparison^22^, as well as the annotators’ own identities and demographics^23^. These factors may help explain why patients rate themselves at scale extremes, while annotators favor mid-range values—suggesting central tendency bias^24^. Future research should consider machine learning-based tools to help mitigate this subjectivity.

Our findings are consistent with existing literature demonstrating variability in skin tone assessment across domain contexts from medicine to computer vision. One group reported moderate internal consistency for the Fitzpatrick Skin Type Scale in a cohort of women undergoing radiation therapy for breast cancer^25^. In the computer vision domain, others found significant inter-rater variability in human Fitzpatrick annotations for facial image with standardized guidelines were provided^26^. Annotation procedures and contextual factors, such as scale presentation order and image context, may significantly impact inter-annotator agreement highlighting the subjectivity in skin tone classification^2^.

Disparities between annotators and patients occurred across both Fitzpatrick and Monk, indicating these challenges are not scale-specific. The strong tendency of patients to rank themselves on the extremes and annotators to score near the center highlights the discrepancies between personal and external perspective in these scales. These findings should push researchers to consider new approaches to enhance the accuracy and consensus in skin tone research.

Using subjective common skin tone scales to assess the validity and accuracies of pulse oximeters specifically, raises concerns about fairness assessment. It is well-described that pulse oximeters may have differential error rates and/or accuracies across different melanin content^5,27–30^. As a result, during COVID-19 surges, allocating resources (e.g., oxygen therapy, disease-modifying pharmacotherapies, ICU-level care) based on hypoxia from pulse oximetry led to inequitable care delivery^31,32^. FDA 510(k) clearances for pulse oximeters have not commonly addressed diverse patient samples and when they do, have increasingly relied on Fitzpatrick scales to demonstrate representation^33^. Following the recent call by the FDA to broaden the evaluation of pulse oximetry across different subjective and objective approaches, it is evident that traditional methodologies, including both the Fitzpatrick and Monk scales, require rigorous reassessment. The FDA has emphasized the importance of using diverse datasets and complementary methods to mitigate bias and improve the reliability of such tools^34^. These challenges extend beyond pulse oximetry. In dermatology inaccurate skin tone classification may lead to misclassification of lesions on darker skin tones, reinforcing structural inequities in care delivery and outcomes^6,7^. Our results also show differences in evaluation of skin tone across both scales at different anatomic sites and across annotators. While using more objective methods to evaluate melanin content is ideal (e.g., spectrophotometry), when using observer-based scales, assessing multiple anatomic regions could minimize variance and having multiple annotators could improve confidence in assessments.

Our study has important limitations. The relatively small sample size, low number of female patients, and predominantly White cohort may affect generalizability. Future studies should broaden patient recruitment to include a wider range of self-reported skin tones. Alternatively, the use of synthetic data could improve validation efforts. Although our annotators represented diverse sociodemographic backgrounds, a larger number of annotators with even more heterogeneity could allow for further analyses on the impact of annotator characteristics on perceived skin tones. Importantly, patients were not provided a mirror or their own pictures to reference during the process, and thus annotators were likely evaluating different skin regions compared to the patients themselves. However, annotator-based evaluations are often performed in this fashion in the real-world and we sought to imitate this approach. Skin tone measures using spectrophotometers or calorimeters could provide more objective measurements, but are infeasible for images in-the-wild or retrospective data. It is also important to reiterate that the Fitzpatrick scale was not originally designed to represent skin color itself, but rather to estimate sun reactivity and risk for UV damage, which may limit its appropriateness for evaluating representation in AI and clinical imaging contexts but is still widely used in the literature. Our study replicated scales commonly used in computer vision, but future work could benefit from incorporating objective melanin measures to better compare skin tone tools. Alternative scales (e.g., Fenty, Pantone) may provide more granularity and consistency across raters and merit further exploration. Annotation lacked environmental standardization, including screen brightness and resolution, which could affect results.

In conclusion, our study highlights a distinct disparity among and between annotator-derived and self-reported skin tone assessments, irrespective of the scale used. This discrepancy calls for caution when using conventional skin tone scales to assess AI fairness and representation. Further research is warranted to develop methods for assessing representation, handling disagreements both within and across annotators, and determining how individual self-reported skin tones should be used when evaluating healthcare AI tools.

## Methods

### Study design

We conducted a prospective observational study of hospitalized adults (≥18 years) at the San Francisco Veterans Affairs Medical Center (VAMC), undergoing surgery (2023–2024). This study was a secondary analysis of a larger trial developing contactless nociception monitors using computer vision^35^. This study was approved by the UCSF IRB (#:13-10913), was performed in accordance with the Declaration of Helsinki. Informed consent was obtained by research participants. We adhered to the Strengthening the Reporting of Observational Studies in Epidemiology (STROBE) statement^36^ (Supplement).

### Participants, image processing, de-identification and attention to privacy

Patients were consented for monitoring using a multi-camera array. Following the video collection, facial images were cropped using RetinaFace^37^. Three distinct facial regions were further isolated using facial landmarks identified by RetinaFace: (1) forehead; (2) left cheek; and (3) right cheek. These areas are commonly used in dermatologic research and regulatory guidance due to visibility, low occlusion risk, and importance in social perception and model performance^34,38,39^. Study team members (JMC, TAH, CC) reviewed images to ensure de-identification.

Each patient provided self-identified sociodemographic information including age, prespecified race and ethnicity classifications within the SF VAMC, and sex. Patients were provided two skin tone scales (Fitzpatrick and Monk) and were asked to self-report their own skin tone based on each scale. The Fitzpatrick skin tone scale ranges from I to VI, while Monk has a range from 1 to 10. Fitzpatrick was selected as it is the most widely used scale to stratify skin tone for computer vision tasks and studies for bias and representation^2,14^. Of note, Fitzpatrick’s original paper^11^ did not include a visual analog scale but it remains one of the most commonly used scales (visually) to assess skin tone. Hence, we implemented a visual scale found online that largely represents visual scales used in the literature^2,8,9,14,15^. We also chose the Monk scale^16^ as it might be able to capture a broader range of skin tones^15^ and because it has been adopted by Google (Alphabet)^17^ for internal bias assessment of their computer vision models.

Patients received printed physical copies of the scales and were instructed to choose the number and associated skin color that best matched their own skin tone (Supplementary Fig. 1a,b). Patients were not instructed on how to self-assess their own skin tones beyond being provided the scales. Surveys were performed in well-lit rooms but lighting varied across hospital units (e.g., PACU, ward, ICU). Lighting conditions were not standardized or measured.

### Annotator assessment procedure

Each image across three facial regions (forehead, right/left cheek) were presented to annotators each in triplicate and at random, using a graphical user interface (GUI) created for this purpose (Fig. 3).

We chose three annotators with diverse ethnic and cultural perspectives, including Hispanic or Latino and Black or African American as self-identified using the NIH race categories and ethnicities^40,41^. All annotations were performed independently and blinded to patient subjective scores, location, facial region and patient characteristics. The GUI presented one image at a time and only one skin tone scale at a time in random sequence to minimize recall bias. This amounted to 18 unique scores per patient per annotator (2 scales, 3 facial locations, each in triplicate). For each annotation, annotators also recorded their self-confidence score using 5-point Likert scale (1-least confident to 5-most confident)^42^. Annotators used their personal computers; display and hardware were not standardized. Annotation results were analyzed by a separate member of the research team who was blinded to the original raw facial images (ND). Data was consolidated into a Pandas dataframe and analyzed in Python (Python Software Foundation.; v3.12^43^). GUI screenshots appear in Supplement (Fig. 2a,b).

#### Statistical analyses

We sought to evaluate internal reliability of annotations across scales, inter-annotator agreement among the annotators and finally to compare annotator differences from subjective skin tone scores. For internal reliability, we used Cronbach’s alpha^44^ across 2 dimensions (1) at a patient-level and (2) at a face location/landmark level. These were performed separately for the Fitzpatrick and Monk scales.

We performed different inter-annotator assessments given the different types of agreement methods that have relative strengths and weaknesses for ordinal classification tasks. Our primary approach was the intraclass correlation coefficient (ICC)^45^ which allows for the evaluation of both the consistency and absolute agreement of the ratings across annotators by accounting for the patient-level and rater-level variability. For ICC, we used a two-way random effects model (ICC[2,k]); this test assumes that both the patients and raters are randomly selected from a larger population providing an estimate of the agreement between the annotators that is generalizable. As sensitivity analyses, we assessed inter-annotator agreement using Kendall’s W^46^, Krippendorff’s alpha^47^, and Weighted Cohen’s Kappa^48^, each with unique abilities in handling ordinal data. Kendall’s W is a non-parametric measure used to capture the relative ranking of skin tone at the patient-level among raters. It is calculated by averaging each annotator’s skin tone rating per patient, ranking patients by these averages for each annotator, and comparing the resulting ordered lists across annotators. Krippendorf’s alpha is a robust inter-annotator agreement metric that accommodates varying sample sizes, missing data, and multiple annotators. Finally, Weighted Cohen’s Kappa allows for partial credit on close agreements, making it valuable in ordinal classifications like skin tone. The weighted matrix penalizes disagreements based on ordinal distance, so minor discrepancies are less penalized than larger ones.

To explore differences between annotators’ and patients’ subjective scores, we assessed whether annotator scores differed from patient self-reported scores using paired *t*-tests^49^. To assess the potential strength and direction of the monotonic relationship between patient self-reported skin tone and annotator’s consensus ratings, we calculated Spearman’s rank correlation^50^. We used a mixed linear model regression adjusting for facial landmark location and annotator confidence to evaluate relationships between annotators’ mean scores and self-reported scores. Bland-Altman Plots^51^ were used to visually represent agreement between annotators and patients. All analyses were performed in Python (Python Software Foundation.; v3.12^43^).

## Supplementary information

Supplementary Information

## Data Availability

Due to patient privacy concerns with personal images and in accordance with institution policy (UCSF), this dataset is not publicly available but the jupyter notebook which analysis was conducted is available via a GitHub repository (https://github.com/ndundas/SkinToneSubjectivity/tree/main). Supplement provides key information on the data analysis pipeline.

## Code availability

Due to patient privacy concerns with personal images and in accordance with institution policy (UCSF), this dataset is not publicly available but the jupyter notebook which analysis was conducted is available via a GitHub repository (https://github.com/ndundas/SkinToneSubjectivity/tree/main). Supplement provides key information on the data analysis pipeline.
