# Supplementary material for: Validity of two subjective skin tone scales and its implications on healthcare model fairness
Source: NPJ Digit Med. 2025 Oct 3;8:595. doi: 10.1038/s41746-025-01975-7 (PMC12494915; doi:10.1038/s41746-025-01975-7)
Supplement: Supplementary file 1 — Supplementary Information [file 41746_2025_1975_MOESM1_ESM.pdf]

Supplement:

	Characteristics
**Total Patients**	n=90
**Age (median years, IQR)**	71.5 (IQR: 58.5, 75.8)
**Sex**	
Male	76.7%
Female	20.0%
Other/Unknown	3.3%
**Race and Ethnicity***	
White	47.8%
Black/African-American	10.0%
Hispanic/Latino	15.6%
Other/Unknown	26.6%
**Native Language**	
English	87.1%
Non-English	12.9%

Supplementary Table 1: SF VAMC patient characteristics used for skin tone measurement

*We followed CMS cell suppression guidelines for patient privacy and the VA Race and Ethnicity categories.

Abbreviations: SFVAMC=San Francisco Veterans Affairs Medical Center; IQR=interquartile range; CMS=Centers for Medicare & Medicaid Services; VA=Veterans Affairs

Scale	Annotator 1	Annotator 2	Annotator 3
**Fitzpatrick**	0.88	0.89	0.92
**Monk**	0.91	0.88	0.93

Supplementary Table 2: Internal Reliability of Annotator Ratings Across Skin Tone Scales

The Fitzpatrick (scale of I–VI) and Monk (scale of 1–10) refer to the two skin tone scales used for this study. Cronbach's alpha values indicating high internal consistency within each annotator for both Fitzpatrick and Monk skin tone scales. Results are presented at the patient level.

Annotator	Scale	Location	Cronbach's Alpha	95% CI
1	Fitzpatrick	forehead	0.82	(0.73, 0.88)
2	Fitzpatrick	forehead	0.88	(0.82, 0.93)
3	Fitzpatrick	forehead	0.91	(0.85, 0.94)
1	Monk	forehead	0.85	(0.76, 0.91)
2	Monk	forehead	0.9	(0.84, 0.93)
3	Monk	forehead	0.9	(0.85, 0.93)
1	Fitzpatrick	left cheek	0.88	(0.83, 0.91)
2	Fitzpatrick	left cheek	0.92	(0.86, 0.95)
3	Fitzpatrick	left cheek	0.86	(0.77, 0.91)
1	Monk	left cheek	0.85	(0.78, 0.9)
2	Monk	left cheek	0.94	(0.86, 0.98)
3	Monk	left cheek	0.84	(0.76, 0.9)
1	Fitzpatrick	right cheek	0.8	(0.7, 0.87)
2	Fitzpatrick	right cheek	0.94	(0.9, 0.97)
3	Fitzpatrick	right cheek	0.91	(0.85, 0.94)
1	Monk	right cheek	0.88	(0.82, 0.92)
2	Monk	right cheek	0.91	(0.84, 0.96)
3	Monk	right cheek	0.9	(0.85, 0.94)

Supplementary Table 3: Reliability Assessment Using Cronbach Alpha by Location
Cronbach's alpha values indicating moderate to high internal consistency within each annotator for both Fitzpatrick and Monk skin tone scales at the location level for each patient.
Abbreviations: CI=Confidence interval

Analysis	Fitzpatrick	Monk
**Paired t-Test (t-statistic)**	3.97 ($p < 0.001$)	6.24 ($p < 0.001$)
**Spearman's Correlation Coefficient**	-0.82	-0.84

Supplementary Table 4: Statistical Comparison Between the Mean Annotator Score and Patient Self-Reported Scores

The t-test was performed between the patient's self-reported scores and the mean of all the annotators scores for each patient. Self-reported and annotated scores were significantly different across both scales. The Spearman's correlation coefficients suggests a strong, inverse monotonic association between self-reported and annotator scores. The negative Spearman correlation suggests (a) higher self-reported scores are associated with lower annotator scores and (b) lower self-reported scores associate with higher annotator scores.

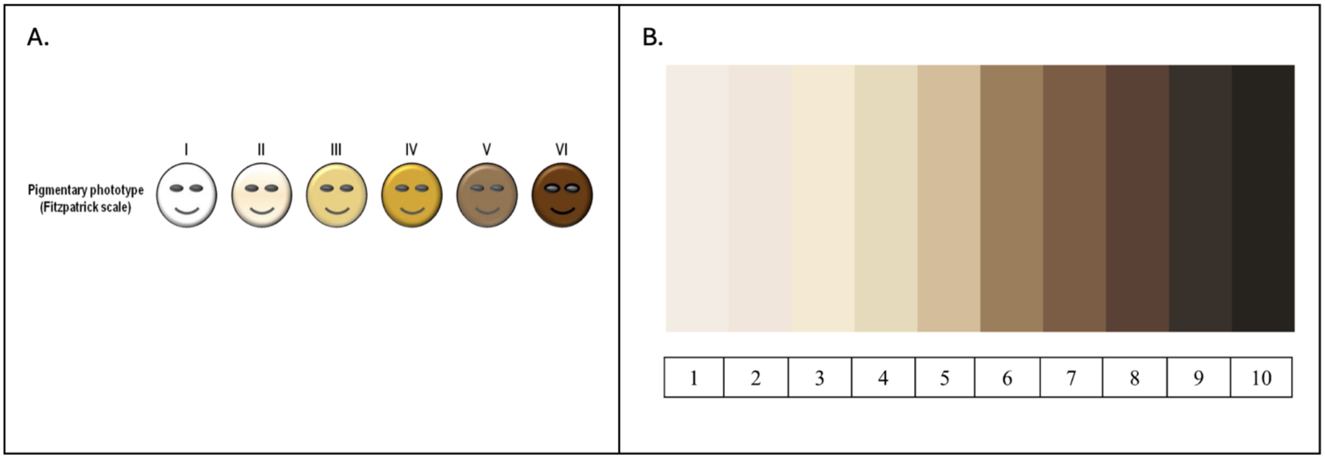

Supplementary Figure 1a-b: Fitzpatrick and Monk Skin Tone Scales
Skin tone is commonly measured using subjective scales called (a) Fitzpatrick and (b) Monk scales. The Fitzpatrick scale was developed in 1975 to identify skin types at heightened risk from ultraviolet radiation and phototherapy and eventually had a visual component (a) that could be used by dermatologists and clinicians. This scale has been widely adopted to classify skin tone and is used to determine representation in biosensor and computer vision studies that incorporate skin of participants. The Monk scale (b) is more recent and created with the intention of being more inclusive, representative measure of skin tones with enhanced diversity.

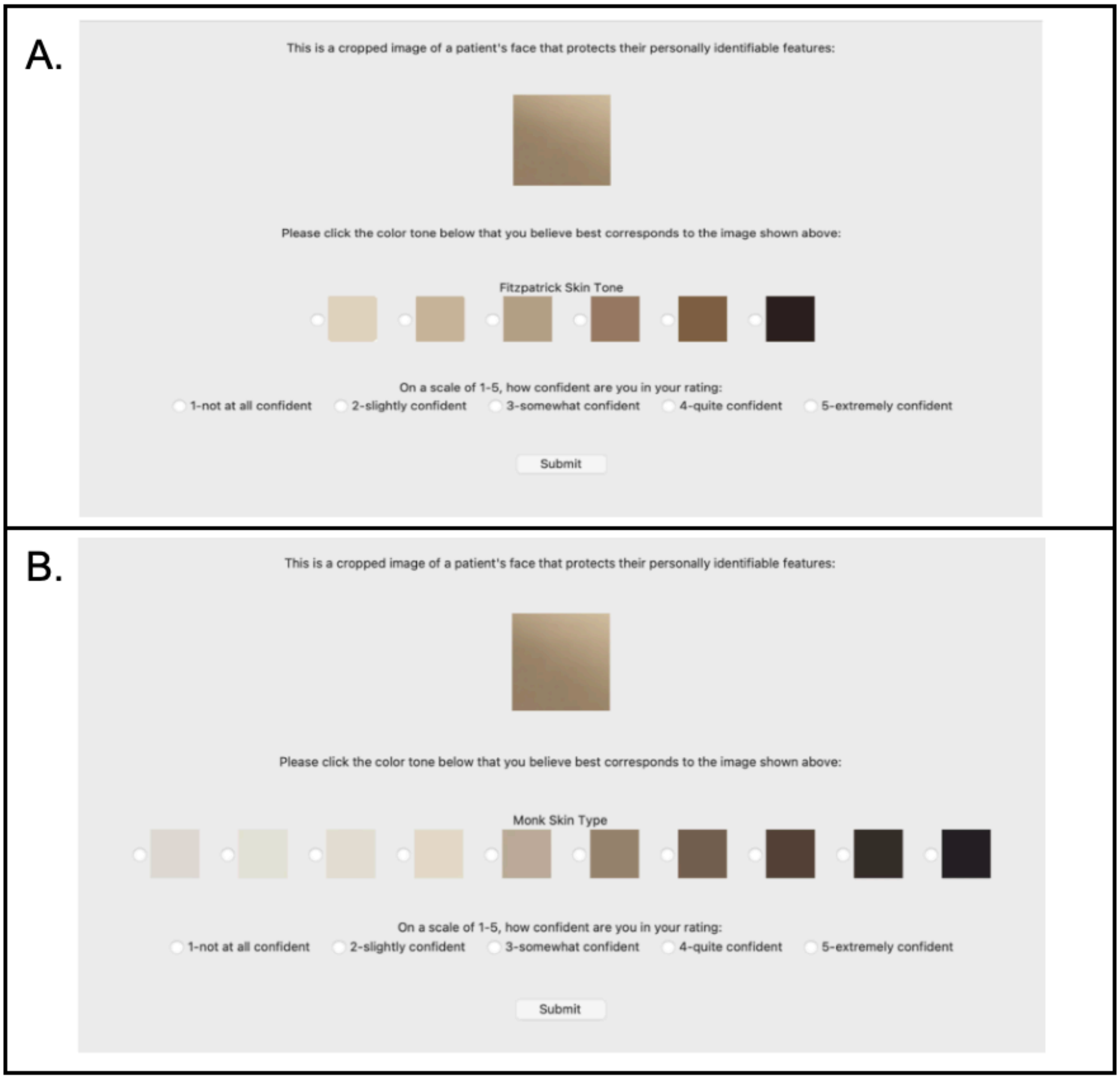

Supplementary Figure 2a-b: Graphical user interfaces (GUI) for annotator ratings of (a) Fitzpatrick and (b) Monk skin tone scales.
Annotators were presented with GUIs that presented a patch of skin from prespecified locations of patients in our cohort at random. GUIs presented only one skin tone scale at a time also at random. No patch of skin was repeated in subsequent GUIs out of concern that previous ratings would inform a subsequent rating for the same patch of skin. All ratings also included a self-reported confidence using a 5-point Likert scale. All GUIs were presented to annotators on a single page. Annotators could not change their votes after the fact. All skin patches were unidentifiable. Annotators were blinded from each other's votes.
Abbreviations: GUI=graphical user interface

a. Fitzpatrick

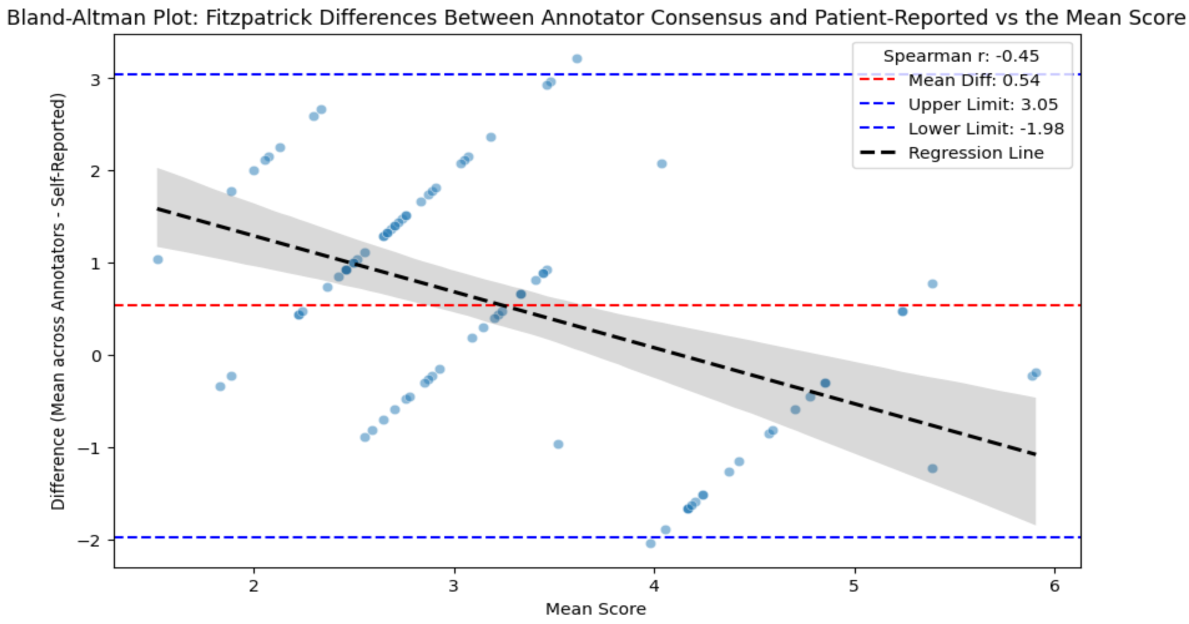

b. Monk

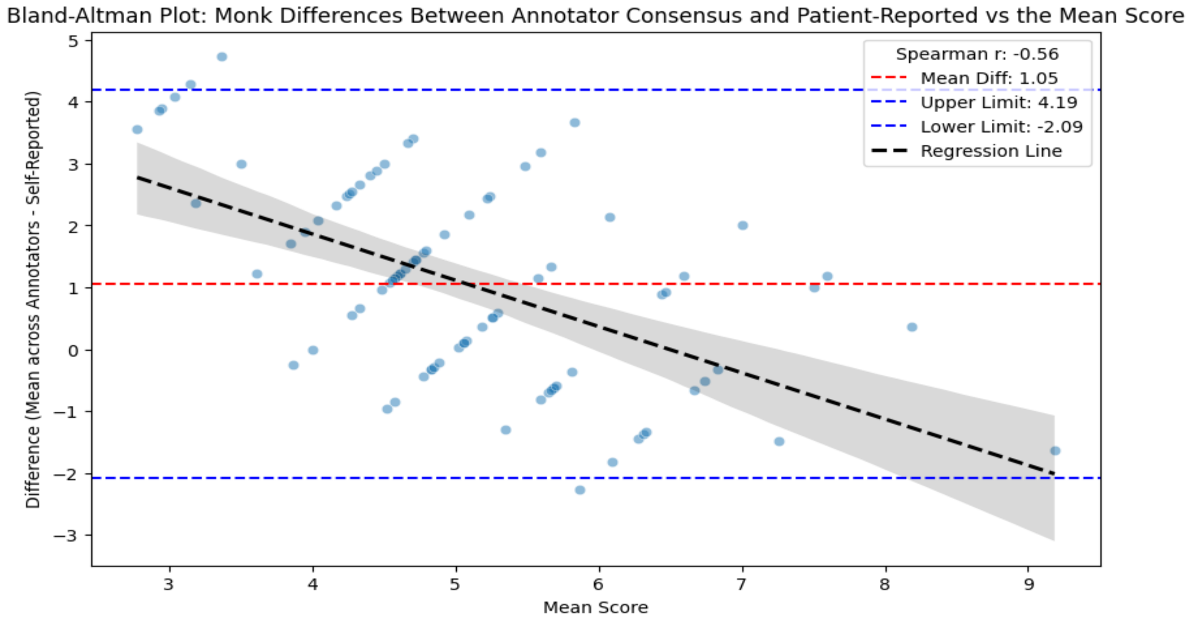

Supplementary Figure 3a-b: Bland-Altman plot comparing (a) Fitzpatrick skin tone ratings and (b) Monk scale between annotators and self-reports.
On average, annotators rated skin tone 0.54 units higher than self-reported values (red dashed line). Most of our data lie within the limits of agreement (-1.98 to 3.05, blue dashed lines). A moderate negative Spearman correlation (-0.45) and the regression line (black dashed) suggest the systematic bias, i.e. annotators tend to overestimate lighter skin tones and underestimate darker ones.

STROBE Statement—Checklist of items that should be included in reports of ***cohort studies***

	Item No	Recommendation	Page No
**Title and abstract**	1	(*a*) Indicate the study's design with a commonly used term in the title or the abstract<br>(*b*) Provide in the abstract an informative and balanced summary of what was done and what was found	1, 4
**Introduction**			
Background/rationale	2	Explain the scientific background and rationale for the investigation being reported	5
Objectives	3	State specific objectives, including any prespecified hypotheses	5
**Methods**			
Study design	4	Present key elements of study design early in the paper	5
Setting	5	Describe the setting, locations, and relevant dates, including periods of recruitment, exposure, follow-up, and data collection	5
Participants	6	(*a*) Give the eligibility criteria, and the sources and methods of selection of participants. Describe methods of follow-up<br>(*b*) For matched studies, give matching criteria and number of exposed and unexposed	6
Variables	7	Clearly define all outcomes, exposures, predictors, potential confounders, and effect modifiers. Give diagnostic criteria, if applicable	6
Data sources/ measurement	8*	For each variable of interest, give sources of data and details of methods of assessment (measurement). Describe comparability of assessment methods if there is more than one group	6
Bias	9	Describe any efforts to address potential sources of bias	6
Study size	10	Explain how the study size was arrived at	-
Quantitative variables	11	Explain how quantitative variables were handled in the analyses. If applicable, describe which groupings were chosen and why	6,7
Statistical methods	12	(*a*) Describe all statistical methods, including those used to control for confounding<br>(*b*) Describe any methods used to examine subgroups and interactions<br>(*c*) Explain how missing data were addressed<br>(*d*) If applicable, explain how loss to follow-up was addressed<br>(*e*) Describe any sensitivity analyses	7
**Results**			
Participants	13*	(a) Report numbers of individuals at each stage of study—eg numbers potentially eligible, examined for eligibility, confirmed eligible, included in the study, completing follow-up, and analysed<br>(b) Give reasons for non-participation at each stage<br>(c) Consider use of a flow diagram	7
Descriptive data	14*	(a) Give characteristics of study participants (eg demographic, clinical, social) and information on exposures and potential confounders<br>(b) Indicate number of participants with missing data for each variable of interest	7

		(c) Summarise follow-up time (eg, average and total amount)	
Outcome data	15*	Report numbers of outcome events or summary measures over time	7,8

Main results	16	(*a*) Give unadjusted estimates and, if applicable, confounder-adjusted estimates and their precision (eg, 95% confidence interval). Make clear which confounders were adjusted for and why they were included<br>(*b*) Report category boundaries when continuous variables were categorized<br>(*c*) If relevant, consider translating estimates of relative risk into absolute risk for a meaningful time period	7,8
Other analyses	17	Report other analyses done—eg analyses of subgroups and interactions, and sensitivity analyses	7,8
**Discussion**			
Key results	18	Summarise key results with reference to study objectives	8
Limitations	19	Discuss limitations of the study, taking into account sources of potential bias or imprecision. Discuss both direction and magnitude of any potential bias	10
Interpretation	20	Give a cautious overall interpretation of results considering objectives, limitations, multiplicity of analyses, results from similar studies, and other relevant evidence	8,9
Generalisability	21	Discuss the generalisability (external validity) of the study results	10
**Other information**			
Funding	22	Give the source of funding and the role of the funders for the present study and, if applicable, for the original study on which the present article is based	2

*Give information separately for exposed and unexposed groups.

Note: An Explanation and Elaboration article discusses each checklist item and gives methodological background and published examples of transparent reporting. The STROBE checklist is best used in conjunction with this article (freely available on the Web sites of PLoS Medicine at http://www.plosmedicine.org/, Annals of Internal Medicine at http://www.annals.org/, and Epidemiology at http://www.epidem.com/). Information on the STROBE Initiative is available at http://www.strobe-statement.org.

CITATIONS:

1. Kendall MG. A NEW MEASURE OF RANK CORRELATION. *Biometrika*. 1938;30(1-2):81-93. doi:10.1093/biomet/30.1-2.81
2. Krippendorff K. *Computing Krippendorff's Alpha-Reliability*.; 2011. http://repository.upenn.edu/asc_papers/43
3. Cohen J. Weighted kappa: Nominal scale agreement provision for scaled disagreement or partial credit. *Psychol Bull*. 1968;70(4):213-220. doi:10.1037/h0026256
